# Comment on Wu et al. Peripheral Biomarkers of Anorexia Nervosa: A Meta-Analysis. *Nutrients* 2024, *16*, 2095

**DOI:** 10.3390/nu17172873

**Published:** 2025-09-05

**Authors:** Johanna L. Keeler, Jonas L. Steinhäuser

**Affiliations:** 1 Centre for Research in Eating and Weight Disorders, Department of Psychological Medicine, Institute of Psychiatry, Psychology & Neuroscience, King’s College London, London SE5 8AF, UK; 2 Else Kröner Fresenius Center for Digital Health, TUD Dresden University of Technology, 01307 Dresden, Germany; jonas.steinhaeuser@ukdd.de; 3 Department of Medicine I, University Hospital Carl Gustav Carus, TUD Dresden University of Technology, 01307 Dresden, Germany; 4 Translational Developmental Neuroscience Section, Division of Psychological and Social Medicine and Developmental Neurosciences, Faculty of Medicine, TUD Dresden University of Technology, 01307 Dresden, Germany

The identification of biomarkers is an important precondition for the development of personalised treatments for anorexia nervosa (AN). We read with great interest the comprehensive meta-analysis by Wu and colleagues [1] examining differences in 52 distinct peripheral molecules between adults with AN and healthy controls and congratulate the authors on this tremendous effort. The authors reported increased concentrations of 10 and decreased concentrations of 15 peripheral molecules in AN compared to controls. Notably, and in contrast to previous meta-analyses [2,3,4], it was found that the peripheral levels of brain-derived neurotrophic factor (BDNF) were not significantly different from those of healthy controls. In this comment, we endeavour to discuss this finding in light of previous research. Overall, the meta-analysis appears to be methodologically robust; however, we would like to highlight two methodological concerns we have identified.

Firstly, one study by Eddy et al. [5] was included twice in the forest plot for the BDNF meta-analysis (Supplementary Materials of original article). Notably, this study had an effect size divergent in direction from the other two studies included in this meta-analysis. According to the Cochrane guidance for meta-analysis, “duplication publication can introduce substantial biases if studies are inadvertently included more than once in a meta-analysis” [6]. Therefore, it is likely that the duplicate inclusion of this study has led to the non-significance of the effect size reported for BDNF.

Secondly, we identified several studies included in previous BDNF meta-analyses [2,3,4] that appear to be eligible for inclusion in the present meta-analysis based on the inclusion and exclusion criteria described by the authors. For example, we found at least two additional publications reporting BDNF concentrations in an adult sample of people with AN and controls [7,8], one of which with mean values and standard deviations that would be available upon request [7]. Additionally, we also noticed additional publications that we believe may have been eligible for inclusion in the meta-analyses of C-reactive protein [9] and lymphocytes [10].

The estimated standardised mean difference when including the study by Eddy et al. [5] increases to *g =* −0.34 (95% CI −0.82, 0.15), and when including the two mentioned additional publications [7,8], it increases further to *g* = −0.59 (95% CI −1.02, −0.16; Figure 1).

In our previous articles and correspondence [13], we highlighted the importance of BDNF as a potential molecule in the pathogenesis of eating disorders and, potentially, as a marker of clinical response to treatment whilst also—and in agreement with the authors—acknowledging that other candidate biomarkers may have more clinical utility (e.g., leptin, IGF-1, gonadal hormones). While this study is overall a strong contribution to the field, we conclude that the meta-analyses would be strengthened by these two methodological considerations to ensure that the broad recommendations of the publication are accurate.

## Figures and Tables

**Figure 1:**
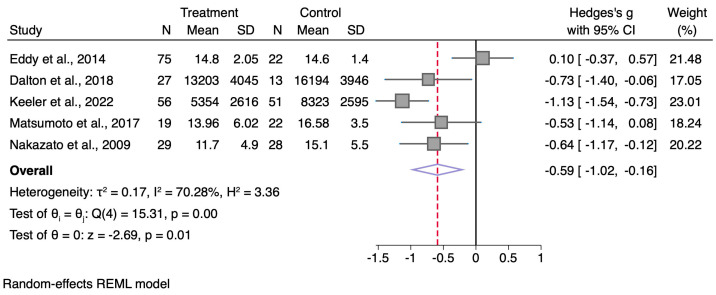
Random-effects meta-analysis with restricted maximum likelihood estimation of BDNF concentrations between adults with AN and healthy controls, using studies included in the publication by Wu et al. [1] and additional identified publications [5,7,8,11,12].

## References

[1] Wu Y.-K., Watson H.J., Del Re A.C., Finch J.E., Hardin S.L., Dumain A.S., Brownley K.A., Baker J.H. (2024). Peripheral Biomarkers of Anorexia Nervosa: A Meta-Analysis. Nutrients.

[2] Brandys M.K., Kas M.J., Van Elburg A.A., Campbell I.C., Adan R.A. (2011). A meta-analysis of circulating BDNF concentrations in anorexia nervosa. World J. Biol. Psychiatry.

[3] Keeler J.L., Robinson L., Keeler-Schaeffeler R., Dalton B., Treasure J., Himmerich H. (2022). Growth factors in anorexia nervosa: A systematic review and meta-analysis of cross-sectional and longitudinal data. World J. Biol. Psychiatry.

[4] Shobeiri P., Bagherieh S., Mirzayi P., Kalantari A., Mirmosayyeb O., Teixeira A.L., Rezaei N. (2022). Serum and plasma levels of brain-derived neurotrophic factor in individuals with eating disorders (EDs): A systematic review and meta-analysis. J. Eat. Disord..

[5] Eddy K.T., Lawson E.A., Meade C., Meenaghan E., Horton S.E., Misra M., Klibanski A., Miller K.K. (2014). Appetite regulatory hormones in women with anorexia nervosa: Binge-eating/purging versus restricting type. J. Clin. Psychiatry.

[6] Lefebvre C., Glanville J., Briscoe S., Littlewood A., Marshall C., Metzendorf M.I., Noel-Storr A., Rader T., Shokraneh F., Thomas J. (2019). Searching for and selecting studies. Cochrane Handbook for Systematic Reviews of Interventions.

[7] Dalton B., Campbell I.C., Chung R., Breen G., Schmidt U., Himmerich H. (2018). Inflammatory markers in anorexia nervosa: An exploratory study. Nutrients.

[8] Keeler J.L., Patsalos O., Chung R., Schmidt U., Breen G., Treasure J., Hubertus H., Dalton B. (2022). Serum levels of brain-derived neurotrophic factor and association with pro-inflammatory cytokines in acute and recovered anorexia nervosa. J. Psychiatr. Res..

[9] Mörkl S., Lackner S., Müller W., Gorkiewicz G., Kashofer K., Oberascher A., Painold A., Holl A., Holzer P., Meinitzer A. (2017). Gut microbiota and body composition in anorexia nervosa inpatients in comparison to athletes, overweight, obese, and normal weight controls. Int. J. Eat. Disord..

[10] Lonati-Galligani M., Pirke K.M. (1986). Beta2-adrenergic receptor regulation in circulating mononuclear leukocytes in anorexia nervosa and bulimia. Psychiatry Res..

[11] Nakazato M., Tchanturia K., Schmidt U., Campbell I.C., Treasure J., Collier D.A., Hashimoto K., Iyo M. (2009). Brain-derived neurotrophic factor (BDNF) and set-shifting in currently ill and recovered anorexia nerosa (AN) patients. Psychol. Med..

[12] Matsumoto J., Hirano Y., Hashimoto K., Ishima T., Kanahara N., Niitsu T., Shiina A., Hashimoto T., Sato Y., Yokote K. (2017). Altered serum level of matrix metalloproteinase-9 and its association with decision-making in eating disorders. Psychiatry Clin. Neurosci..

[13] Steinhäuser J.L., Wronski M.L., Keeler J.L., Ehrlich S., King J.A. (2022). Barking up the wrong biomarker? Correspondence to Shobeiri et al. (2022) “Serum and plasma levels of brain-derived neurotrophic factor in individuals with eating disorders (EDs): A systematic review and meta-analysis”. J. Eat. Disord..

